# Dietary and metabolic effects on the oral status of patients with phenylketonuria: a nation-based cross-sectional study

**DOI:** 10.1007/s00784-022-04827-w

**Published:** 2023-02-20

**Authors:** Anne Carolin Bingöl, Memduh Bingöl, Nikolaos Pandis, Simone Stolz, Skadi Beblo, Paul-Georg Jost-Brinkmann, Eberhard Mönch, Theodosia Bartzela

**Affiliations:** 1 Department of Orthodontics and Dentofacial Orthopedics, Charité – Universitätsmedizin Berlin, corporate member of Freie Universität Berlin and Humboldt-Universität Zu Berlin, Institute for Oral Health Sciences, Aßmannshauser Str. 4-6, 14197 Berlin, Germany; 2 Department of Orthodontics and Dentofacial Orthopedics, Dental School/Medical Faculty, University of Bern, Freiburgstr. 7, 3010 Bern, Switzerland; 3 Department of Pediatric and Adolescent Medicine, Carl-Thiem-Klinikum Cottbus, Thiemstr. 111, 03048 Cottbus, Germany; 4 Department of Women and Child Health, Centre for Pediatric Research Leipzig, Hospital for Children and Adolescents, University of Leipzig, Liebigstr. 20a, Haus 6, 04103 Leipzig, Germany; 5 Interdisciplinary Metabolism Centre, Charité – Universitätsmedizin Berlin, corporate member of Freie Universität Berlin and Humboldt-Universität Zu Berlin, Campus Virchow-Klinikum, Augustenburger Platz 1, 13353 Berlin, Germany; 6 Department of Orthodontics, Technische Universität Dresden, Fetscherstraße 74, 01307 Dresden, Germany

**Keywords:** Phenylketonuria, Oral status, Caries, Enamel defects, Oral health

## Abstract

**Objectives:**

The aim of this study was to compare the prevalence of oral diseases (caries, periodontal disease, enamel defects) between patients with phenylketonuria (PKU), their siblings, and a matched control group.

**Materials and methods:**

A total of 109 patients with PKU, 14 siblings of PKU patients, and 100 healthy individuals aged 6 to 68 years were recruited. All participants completed a questionnaire based on their health status. The patients’ decayed/missing/filled teeth index (dmft/DMFT), gingival bleeding index (GBI), plaque control record (PCR), periodontal screening and recording index (PSR), and developmental enamel defects index (DDE) were recorded. Descriptive statistics and regression modeling were used to examine potential associations between the exposure and the outcomes of interest.

**Results:**

Patients with PKU had 1.6 times more caries (95% confidence interval (CI) 1.22 to 2.20; *p* = 0.001), seven times more enamel defects (95% CI 3.94 to 14.21; *p* < 0.001), and four times higher PSR values (95% CI 2.26 to 7.15; *p* < 0.001) than the control group. The siblings had significantly fewer enamel defects but no significant differences in caries and periodontal parameters compared to the PKU patients.

**Conclusions:**

The results showed a higher risk for the development of caries, periodontitis, and enamel defects in PKU patients.

**Clinical relevance:**

Implementation of preventive measures and regular dental care is necessary for patients with PKU.

## Introduction

Phenylketonuria (PKU) is a rare (1:10,000 newborns in Europe) [1], monogenic, and autosomal recessive inherited metabolic disease associated with a lifelong radically restrictive diet. The classic PKU is associated with more than 950 mutations in both alleles of the *PAH* gene, encoding the enzyme phenylalanine hydroxylase (PAH), which converts phenylalanine (PHE) into tyrosine [2]. Patients with PKU have reduced PHE metabolism. Consequently, PHE is accumulated in the blood and brain at toxic levels. Untreated PKU causes severe mental retardation, epilepsy, and behavioral problems [3, 4]. For this reason, PKU screening is a standardized procedure for newborns in many countries, including Germany [5–7]. Key therapeutic tools include a PHE–restricted diet [8–10] and regular monitoring of blood PHE levels from birth [4, 10, 11].

The dietary management consists of natural protein restriction tailored to the patient’s individual PHE tolerance, a PHE-restricted diet with specific low-protein foods, and daily intakes of PHE-free protein substitutes [10, 12].

PHE is an essential amino acid found in most intact protein sources in food [13]. Therefore, the diet of affected individuals should include low-protein natural foods such as fruits, vegetables, sugars, fats, and industrially produced special low-protein foods such as special bread and pasta [4, 14].

Patients with PKU must measure and count the PHE exchange daily following their individual PHE tolerance [12].

Dietary intake in these patients consists PHE-free protein substitutes three to four times daily to prevent protein deficiency and optimize metabolic control [15]. These tasteless acid concoctions are often ingested with sugary drinks to improve the taste [16].

Patients with PKU are potentially more susceptible to caries than healthy individuals because of their sugar- and carbohydrate-rich diet.

Developmental defects of enamel (DDE) are complications of enamel mineralization, affecting the primary or permanent dentition [17].

The underlying causative mechanism of DDE remains inconclusive [17, 18]. Systemic (maternal diseases, hypoxia, cesarean section, premature birth, early childhood illnesses) and epigenetic/genetic factors may act synergistically or additively on the occurrence of DDE [19–22].

It has been concluded that PKU increases the DDE risk [23, 24].

To the best of our knowledge, no studies have assessed periodontal disease in patients with PKU until now [23, 25, 26]. The existing evidence on the association between PKU and oral indices is limited and often contradictory [23, 25–28].

Therefore, the primary objectives of this study were as follows: (1) to determine the prevalence of oral diseases (caries, periodontal disease, enamel defects) between patients with PKU, their siblings, and a matched non-affected (control) group and (2) to identify predictors of the clinical parameters.

## Materials and methods

The present study has obtained ethical approval from the ethics committees of the Charité – Universitätsmedizin Berlin (EA2/036/18), where the study was initiated, and the University of Leipzig (369/18–lk).

The principles of the Declaration of Helsinki concerning research involving human participants were followed. The study was registered in the German Clinical Trials Register and International Clinical Trials Registry Platform (DRKS00027482).

This is a multicenter cross-sectional study analyzed like a case–control study by comparing the odds of exposure between groups with and without the disease [29].

### Patient selection

The study recruited patients with PKU from nine specialized metabolic disease outpatient clinics in Germany. An attempt was made to include as many patients’ siblings as possible. It was assumed that siblings have similar oral health status and behavior due to the same parental home.

The healthy subjects were selected from the Charité **–** Universitätsmedizin Berlin (Charité Center for Oral Health Sciences CC 3) and a private dental practice (Charlottenburg, Berlin).

The inclusion criteria were as follows: (1) patients with PKU on PHE-restricted diet, (2) past or current use of PHE-free protein substitutes, and (3) at least six years of age (early mixed dentition). The control group included healthy individuals matched in sex and age with the PKU group.

### Methods

One dentist (ACB) performed the oral examination during routine patient recall visits in the PKU specialized centers or dental clinics or during educational events for PKU patients.

Participants or caregivers filled out a questionnaire developed especially for this study on patient health status, dietary habits, oral hygiene, and dental treatment. The questionnaires’ collected information was summarized and used as predictors for the oral indices. Furthermore, the following information from medical records of patients with PKU was registered: the highest PHE level during newborn screening, treatment initiation, protein substitutes, and PHE tolerance per day.

The patient, in the case of an adult participant or the patient’s guardian, signed the consent form. The participant’s information sheet was formulated separately for each age group (children, adolescents, and adults).

The dentist wore a headlamp and magnifying glasses and used a dental mirror and a WHO (World Health Organization) periodontal probe for the dental screening of each subject. Patients were examined in a regular sitting chair available in the examination area.

The decayed/missing/filled teeth index for permanent and mixed dentition (dmft/DMFT) was used to determine the caries experience [30].

The recommended protocol of the WHO for oral health surveys is based only on clinical examinations and excludes dental radiographs [31].

The gingival bleeding index (GBI) [32], plaque control record (PCR) [33], periodontal screening, and recording index (PSR) [34] were used to evaluate the gingival health, oral hygiene, and periodontal status of the patients.

The DDE was used to describe the enamel’s quantitative or qualitative possible alterations [35]. All enamel defects were recorded using the following classification system: absent (average condition), demarcated opacities, diffuse opacities, and hypoplasia. If defects did not fall into these categories, they were scored as “other” [24, 35].

The study protocol has not changed despite the COVID-19 pandemic. The diagnostic tools, a dental mirror, and a dental probe remained the same. The surgical masks were replaced by the FFP2 masks during the examination procedures. Parental presence was allowed depending on the patient’s age. Patients and parents had to provide a negative COVID-19 test for their routine examination in the center. During the registration of the control group, the national recommendations for emergency dental treatment only were followed.

### Method error

Before the participants’ dental examination, calibration sessions between examiners for all oral indices of fifteen patients took place (ACB; TB) to assess the interobserver variability. All results were in 100% agreement. Twenty-one patients were reexamined by ACB for the assessment of the intraobserver variability. The intraobserver duplicate measurement error was calculated using kappa statistics. Kappa is 0.82 for the dmft/DMFT index and 0.64–1.00 for the DDE value.

### Statistical analysis

The Institute of Biometry and Clinical Epidemiology of the Charité determined the sample size calculation. Under the assumption that the dmft/DMFT in the PKU group would be 0.6 and 1.0 in the control group and the standard deviation is 1.6 [23] (relative effect size *d* = 0.4), at least *n* = 100 patients per group were required. Descriptive statistics were calculated. Negative binomial regression (dmft/DMFT), ordered logistic regression (PSR, highest PSR value of all sextants), and logistic regression (DDE), adjusted for age, were used for statistical comparisons between PKU patients, control group, and siblings. Predictors for the oral indices dmft/DMFT, PSR, and DDE were determined by ordered logistic regression. A generalized estimating equation (GEE) population-averaged model was used to examine the effect of tooth segment on DDE after adjusting for the PKU and control group, siblings, and age.

The significance level was set at 5% (*p* = 0.05).

All statistical analyses were performed using SPSS, version 26.0 (IBM Corp SPSS Statistics, Armonk, NY, USA) and the Stata statistical software package (version 16.1; StataCorp, College Station, TX, USA).

## Results

From September 2018 to July 2020, 109 patients with PKU, 14 siblings, and 100 healthy individuals as a control group were recruited from 9 different centers in Germany (Table 1). At the beginning of the COVID–19 pandemic outbreak (22.03.2022–04.05.2022), no patients were recruited.

**Table 1:** Distribution of patients with PKU, patients’ siblings, and control group according to the recruiting center

	Center	Prevalence	Percent
PKU (*N* = 109)	Berlin^a^	39	35.8
	Cottbus	26	23.9
	Leipzig	21	19.3
	Magdeburg	7	6.4
	Rostock	5	4.6
	Hamburg	4	3.7
	Schwerin	2	1.8
	Neuss	3	2.8
	Hannover	1	0.9
	missing	1	0.9
Control (*N* = 100)	Berlin^bc^	100	100.0
Siblings (*N* = 14)	Berlin^a^	2	14.3
	Cottbus	6	42.9
	Leipzig	6	42.9

PKU, phenylketonuria; N, total number of participants in each group; Berlin^a^, Charité – Universitätsmedizin Berlin, Campus Virchow-Klinikum; Berlin^b^, Charité – Universitätsmedizin Berlin, Institute for oral health sciences CC 3; Berlin^c^, private dental practice (Charlottenburg, Berlin); Cottbus, Carl – Thiem Hospital of Cottbus; Leipzig, Magdeburg, Rostock, the University Hospitals of Leipzig, Magdeburg, and Rostock

The mean age for children and adolescents/adults is 11.4 and 31.4 years for the PKU patients, 11.1 and 29.3 years for the control group, and 10.2 and 41 years for the siblings of the PKU patients, respectively (Table 2).

**Table 2:** Participants’ distribution, mean age, standard deviation, and age range of patients with PKU, their siblings, and the control group

Age	PKU patients (*N* = 109)		Control (*N* = 100)		Siblings of PKU patients (*N* = 14)	
	Child (*N* = 59)	Adult (*N* = 50)	Child (*N* = 50)	Adult (*N* = 50)	Child (*N* = 12)	Adult (*N* = 2)
Mean age	11.41	31.42	11.14	29.30	10.17	41.00
sd	3.40	10.64	3.13	7.52	3.16	0.00
Age range	6–17	18–68	6–17	18–54	6–17	41

PKU, phenylketonuria; N, total number of participants in each group; sd, standard deviation

### Dental caries

The dmft/DMFT Index showed a mean (standard deviation) of 5.61 (6.55) for the group with PKU, 4.43 (5.95) for their healthy siblings and 2.84 (3.27) for the control group (Table 3).

**Table 3:** PSR and dmft/DMFT index for PKU patients, control group, and siblings of the PKU patients

	dmft/DMFT				PSR			
	Minimum	Maximum	Mean	Standard deviation	Minimum	Maximum	Mean	Standard deviation
PKU (*N* = 109)	0	28	5.61	6.55	0	4	1.99	0.95
Control (*N* = 100)	0	13	2.84	3.27	0	3	1.52	0.63
Siblings (*N* = 14)	0	18	4.43	5.95	1	3	1.64	0.84

dmft/DMFT, decayed/missing/filled teeth; PSR, periodontal screening and recording index; PKU, phenylketonuria; N, total number of participants per group

**Fig. 1:**
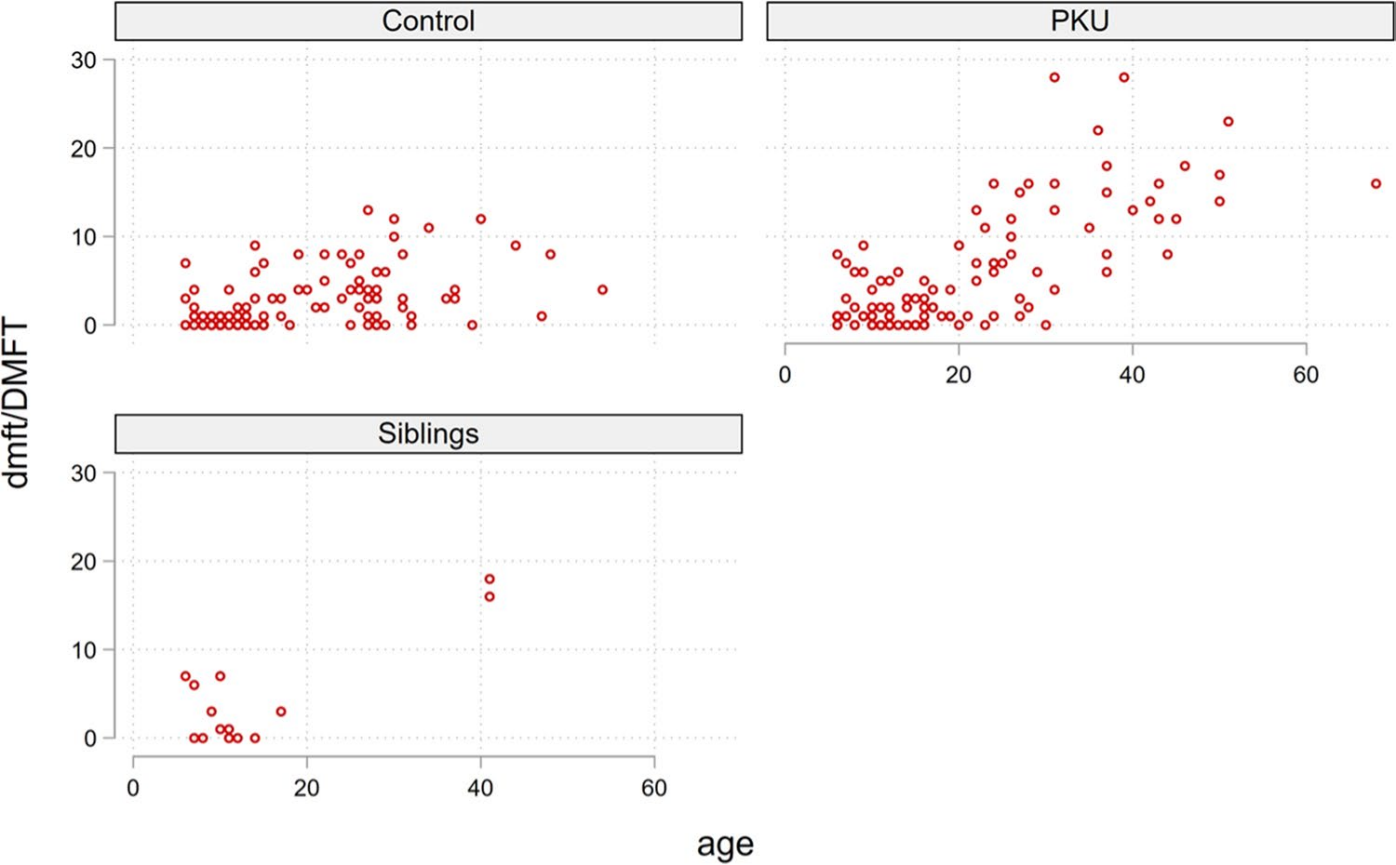
Scatter diagram of dmft/DMFT by age (in years) in the control, PKU, and sibling groups *dmft/DMFT*, decayed/missing/filled teeth; *PKU*, phenylketonuria

The scatter diagram (Fig. 1) shows that dmft/DMFT levels increased with age in the PKU group and are more scattered than in the control group. There were two adult outliers with very high DMFT values among the siblings.

The dmft/DMFT increased with age; every additional year, there was a mean increase in dmft/DMFT by 6% in all groups (Table 4). After adjusting for age as a continuous variable, the patients with PKU had 1.64 times higher caries incidence than the healthy control group (*p* = 0.001). The siblings had 1.84 times higher caries incidence than the control group (*p* = 0.052).

**Table 4:** Negative binomial regression model of dmft/DMFT, ordered logistic regression model of highest sextant PSR value per subject, and logistic regression model of DDE of the control group, PKU, and siblings, adjusted for age (years)

	dmft/DMFT			PSR			DDE		
	IRR	*p*	[CI]	OR	*p*	[CI]	OR	*p*	[CI]
**Control**	Ref			Ref			Ref		
**PKU**	1.64	**0.001**	[1.22, 2.20]	4.02	** < 0.001**	[2.26, 7.15]	7.48	** < 0.001**	[3.94, 14.21]
**Siblings**	1.84	0.052	[1.00, 3.42]	2.60	0.110	[0.80, 8.41]	0.91	0.896	[0.23, 3.64]
**Age**	1.06	** < 0.001**	[1.05, 1.07]	1.15	** < 0.001**	[1.12, 1.19]	0.97	**0.020**	[0.94, 0.99]

dmft/DMFT, decayed/missing/filled teeth; PSR, periodontal screening and recording index; DDE, developmental enamel defects index; IRR, incidence rate ratios for the negative binomial regression model; CI, 95% confidence interval; OR, odds ratio for the ordered logistic and logistic regression; Ref., reference/ base

**Fig. 2:**
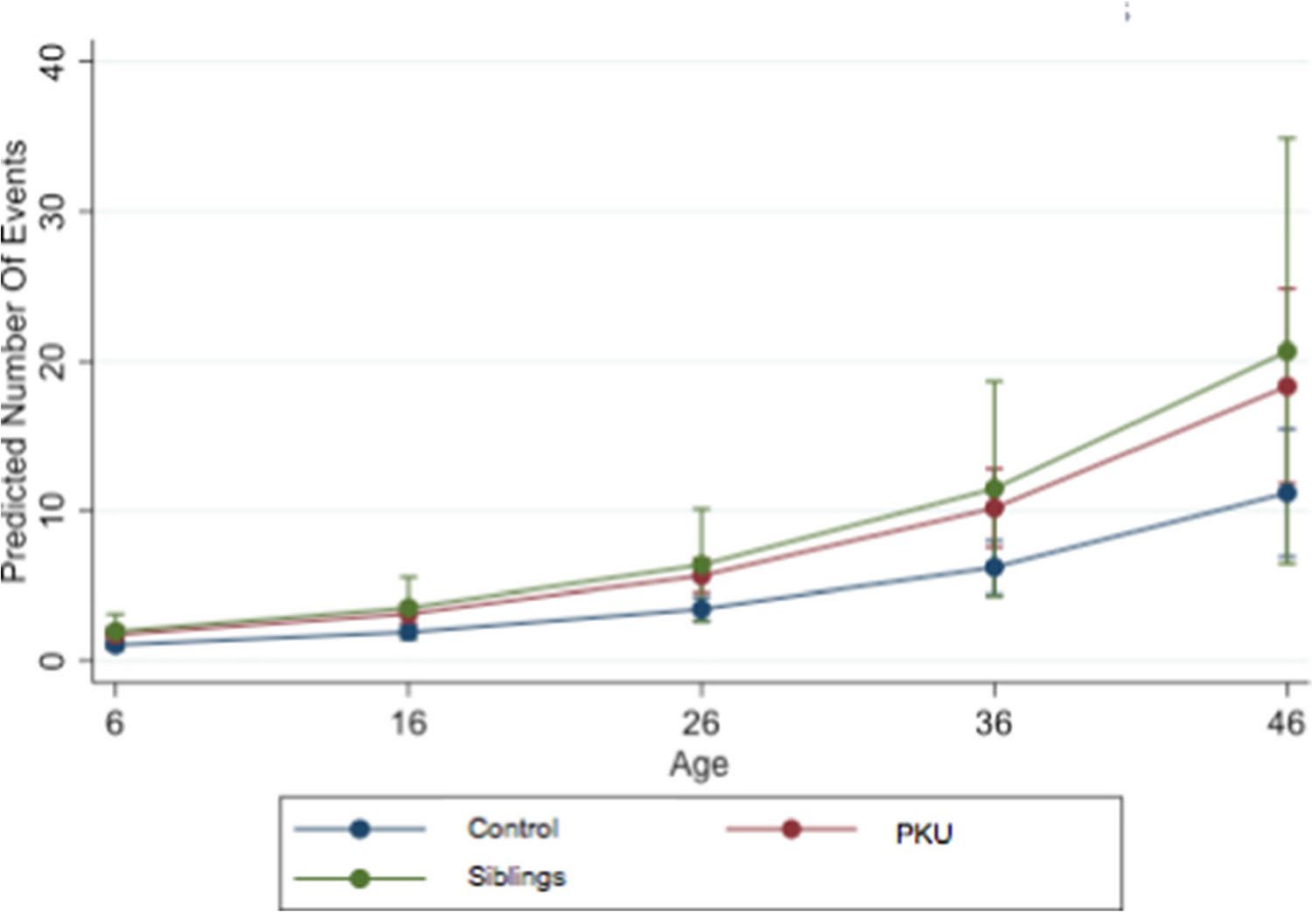
Predicted dmft/DMFT index (number of events) by age and group control, PKU, and siblings; *dmft/DMFT*, decayed/missing/filled teeth; predicted number of events, increase of dmft/DMFT; age, in years; PKU, phenylketonuria

Figure 2 shows the predicted number of events per patient group in 10-year increments.

The variables (current phenylalanine tolerance, meals, and sweet meals per day, frequency of tooth brushing per day, fluoride supplementation, mouth rinsing/gels or varnishes, flossing, and the number of annual dental visits) were not significant predictors of caries risk in this study. One hundred percent of the siblings and control group and 97.3 percent of the PKU patients regularly used fluoridated toothpaste.

### Oral hygiene status and periodontal screening

The mean of the highest sextant PSR value per subject was 1.99 (0.95) for the PKU patients, 1.64 (0.84) for their siblings, and 1.52 (0.63) for the control group (Table 3).

**Fig. 3:**
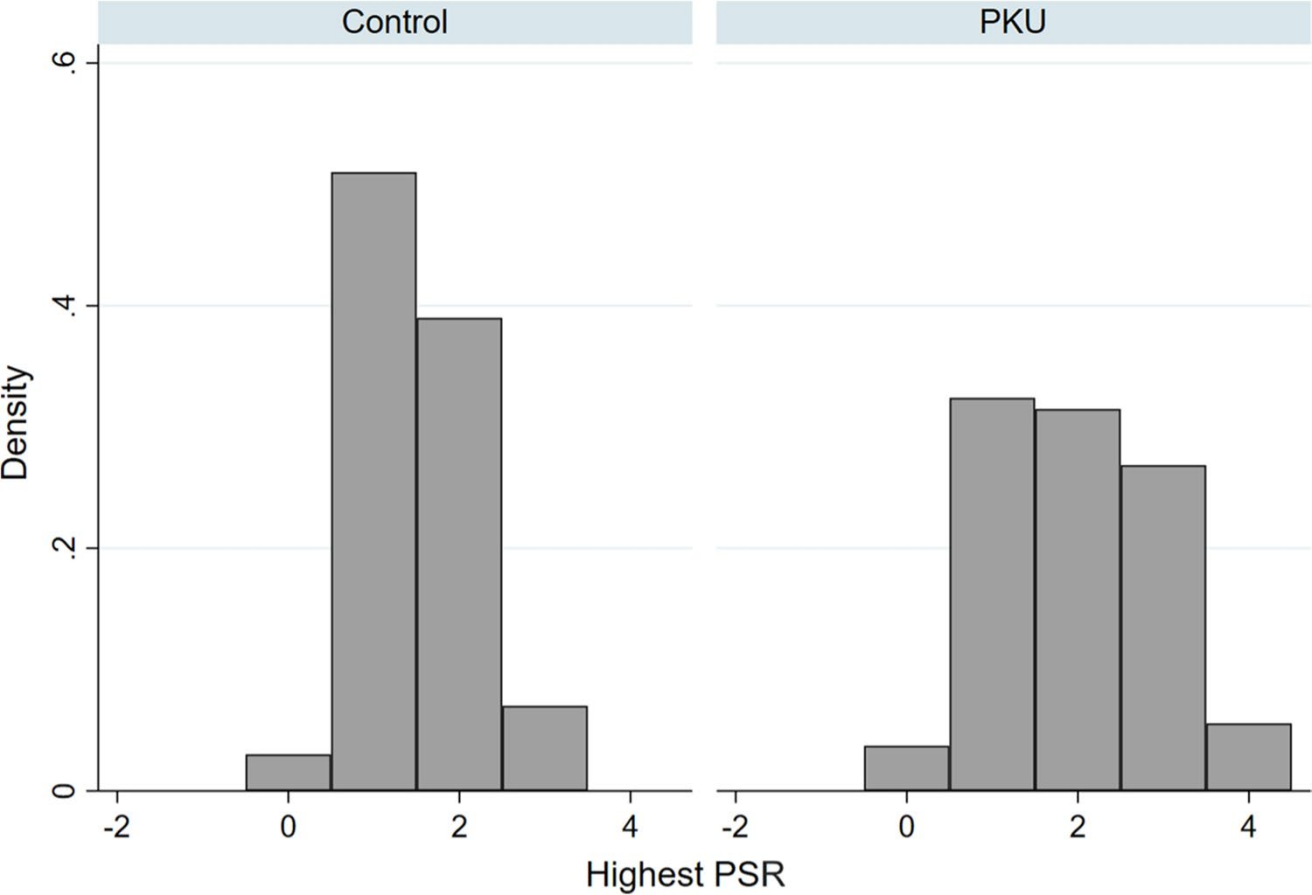
Histograms of the highest PSR value of the PKU patients and the control group PKU, phenylketonuria; highest PSR, highest periodontal screening and recording value per patient

The PKU group was more often scored with codes 3 or 4, indicating the severity of the periodontal disease. The control group displayed scores of 1 or 2, whereas a score of 4 was not recorded in any participant (Fig. 3).

After adjusting for age as a continuous variable, the odds of higher PSR values were 4.02 times higher in the PKU compared to the control group (*p* < 0.001) (Table 4). The siblings had 2.6 times higher odds for greater PSR values than the control group (*p* = 0.11). Every year, the odds of belonging to a higher PSR category increased on average by 15% across all groups.

Significant predictors of the PSR index in this study were increased GBI (*p* < 0.001) and PCR (*p* < 0.001). After adjusting for age as a continuous variable, PKU patients showed three times higher GBI (*p* < 0.001) compared to the control group with regard to the PSR (Table 5). Factors such as smoking, medication, blood count changes, dental visits per year, and dental hygiene (flossing, toothbrushing, mouth rinsing solution, etc.) were not significant predictors. The smoking rate was 7% each for PKU and siblings and 14% for the control group.

**Table 5:** Ordered logistic regression of predictors GBI and PCR of highest sextant PSR value of PKU and control group, adjusted for age (years)

Highest PSR	OR	*p*	[CI]
GBI	1.12	** < 0.001**	[1.06, 1.18]
Control	Reference		
PKU	2.96	** < 0.001**	[1.62, 5.41]
Age	1.13	** < 0.001**	[1.10, 1.17]
PCR	1.13	** < 0.001**	[1.09, 1.17]
Control	Reference		
PKU	1.83	0.065	[0.96, 3.46]
Age	1.16	** < 0.001**	[1.12, 1.20]

PSR, periodontal screening and recording index; GBI, gingival bleeding index; PKU, phenylketonuria; PCR, plaque control record; OR, odds ratio; CI, 95% confidence interval

### Enamel defects

Among the participants, the prevalence of DDE detected was 63.9%, 21.4%, and 19% for the PKU, siblings, and control group, respectively. The PKU group had significantly higher odds (7.48 times) of DDE than the control group (*p* < 0.001), whereas the siblings had 0.91 times lower odds for DDE compared to the control (*p* = 0.90) (Table 4).

The odds of DDE were significantly (*p* < 0.001) lower for all segments compared to the segment of first molars and incisors in the maxilla (Table 6). Age was not significant, suggesting that PKU status and segments account for the outcome.

**Table 6:** Adjusted estimates, 95% confidence intervals, and *p***-**values from the generalized estimating equation (GEE) model for the effect of segments on DDE after adjusting for PKU group, control, siblings, and age (in years)

Tooth segments	OR	*P*	[CI]
max6_2_2	Reference		
max3_4_5	0.36	** < 0.001**	[0.28, 0.47]
mand6_2_2	0.28	** < 0.001**	[0.22, 0.35]
mand3_4_5	0.19	** < 0.001**	[0.14, 0.24]
all7s	0.16	** < 0.001**	[0.12, 0.22]
Control	Reference		
PKU	4.19	** < 0.001**	[2.19, 8.02]
Siblings	1.11	0.891	[0.24, 5.22]
Age	0.98	0.183	[0.96, 1.01]

OR, odds ratio; CI, 95% confidence interval; PKU, phenylketonuria; max6_2_2, segment of the teeth 16, 12, 11, 21, 22, 26; max3_4_5, segment of the teeth 15, 14, 13, 23, 24, 25; mand6_2_2, segment of the teeth 36, 32, 31, 41, 42, 46; mand3_4_5, Segment of the teeth 35, 34, 33, 43, 44, 45; all7s, Segment of the teeth 17, 27, 37, 47; The FDI World Dental Federation notation system for teeth numbering was used

**Fig. 4:**
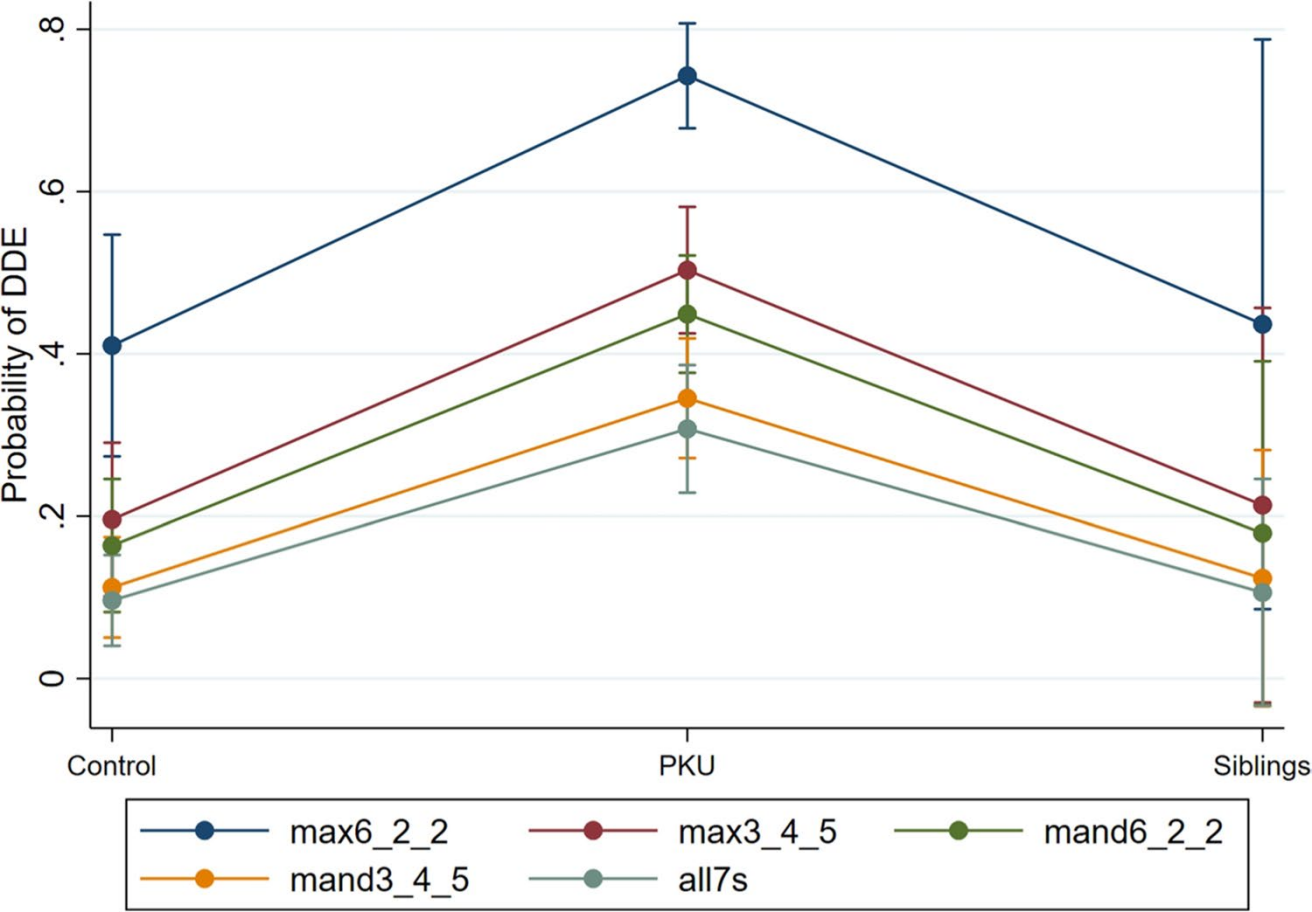
Plot shows the probabilities of DDE by tooth segment and patients with PKU, control group, and siblings. DDE, developmental enamel defects index; PKU, phenylketonuria; max6_2_2, segment of the teeth 16, 12, 11, 21, 22, 26; max3_4_5, segment of the teeth 15, 14, 13, 23, 24, 25; mand6_2_2, segment of the teeth 36, 32, 31, 41, 42, 46; mand3_4_5, segment of the teeth 35, 34, 33, 43, 44, 45; all7s, segment of the teeth 17, 27, 37, 47. The FDI World Dental Federation notation system for teeth numbering was used

Figure 4 shows the predicted probability of DDE was the highest in PKU patients.

There was no significant association between the questionnaire-based predictors (number of annual dental visits, highest PHE value in newborn screening, start of PKU treatment) and the exposure groups.

## Discussion

In this study, we presented the oral status of PKU patients, a control group of healthy individuals, and a small group of non-affected siblings of the PKU group.

Patients with PKU have a significantly higher risk of developing caries, periodontal disease, and enamel defects than unaffected controls.

PKU is a rare disorder [1], and thus patient recruitment is difficult. We recruited 109 patients with PKU, including children, adolescents, and adults, from a German healthcare network (Table 1).

Previous studies presented oral health and disease data in patients with PKU ranging between 24 and 41 subjects. Moreover, only children and adolescents were encompassed in those studies [23–26].

To our knowledge, this study has the most PKU participants, and it is the first study to present the oral health status of children/adolescents and adults with PKU filling knowledge gaps on age-related parameters.

In an ideal research setting, the control group would come from the exact location as the experimental group. Organizational restrictions made this criterion challenging to implement. Hence, the control group was from the university clinic and a dental practice in Berlin.

### Dental caries

Our study shows that patients with PKU had significantly more caries than healthy individuals at any age (Fig. 1 and 2). Contrarily, other studies found no significant difference in caries prevalence between PKU patients and the control group [23, 25], while others described a lower [27] or higher [26, 28] caries prevalence in children with PKU.

In one of the studies with no difference in caries prevalence, 18 of 41 children with PKU received fluoride regularly, while only two of 41 healthy control children received fluoride [23]. The unequal fluoride intake of the experimental and control group make these results inconclusive [36, 37]. Therefore, it is crucial to filter the risk factors associated with caries and correlate them with the results.

Other researchers reported that children with PKU have significantly more caries in the primary dentition but not in the permanent [26]. In the previously reported study [26] and our study, all subjects had a relatively equal fluoride intake. Some inconsistencies in the published data may be attributed to the included groups’ age–related differences. It is hard to compare PKU patients of 8.5 years of average age and a control group of 10.5 years because the permanent teeth newly erupted. Therefore, these teeth have a lower caries risk at a younger age [26]. We included adults in the sample for a more suitable assessment of caries risk in permanent teeth.

Only 14 siblings could be recruited in this study. The siblings had a similar caries index as the PKU patients and a much higher one than the control group. Two of the siblings and one of the PKU patients had a motoric impairment and used a wheelchair. These patients had very high DMFT levels (Fig. 1). Due to the small number of siblings and the two outliers, the results may be skewed. On the other hand, parents of PKU patients carry a high burden and responsibility regarding their children's diet and medical attention, which could lead to somewhat neglect of the oral health of the siblings.

### Oral hygiene status and periodontal screening

This is the first study to screen patients with PKU using the PSR index, a comprehensive periodontal evaluation tool for the clinical detection and documentation of the presence and extent of periodontal diseases endorsed by the American Academy of Periodontology [34, 38]. The study demonstrates that patients with PKU have significantly higher PSR levels and, thus, an increased risk of periodontitis compared to healthy individuals. Predictors of increased PSR in this study are increased PCR (bacterial plaque accumulation) and GBI (gingival inflammation), which have commonly been shown to play a major role in the development of periodontal disease [39–41]. PSR codes 1 and 2 indicate plaque-associated gingivitis, code 3 moderate periodontitis, and code 4 severe chronic periodontitis and warrants further periodontal evaluation [42]. Patients with PKU were found more often to have code 3 or 4. The control group displayed code 1 or 2, whereas code 4 did not appear in this study (Fig. 3).

In conclusion, patients with PKU need further periodontal treatment more often. Control of the periodontal biofilm with professionally administered oral hygiene can slow down or treat periodontitis and prevent tooth loss for many years [39].

Smoking increases the chance of developing periodontitis and is associated with higher levels of periodontal destruction [43] but at the same time reduces the inflammatory response to dental plaque in the gingiva [44]. Although patients with PKU showed a higher PSR and GBI, they smoked less than the control group.

### Enamel defects

The results of this study prove that patients with PKU (63.9%) had significantly more enamel defects compared to their siblings (21.4%) and healthy individuals (19.0%).

In line with our study, de Marco Salvadori et al. analyzed two groups with 24 subjects each, with and without PKU, and found that patients with PKU had significantly more DDE (36%) than the healthy control group (15%). The upper incisors and first molars were also most frequently affected (Fig. 4) [24]. In another study, a significantly greater number of developmental defects were found only in the permanent teeth in the PKU group (*n* = 41) [23].

**Fig. 5:**
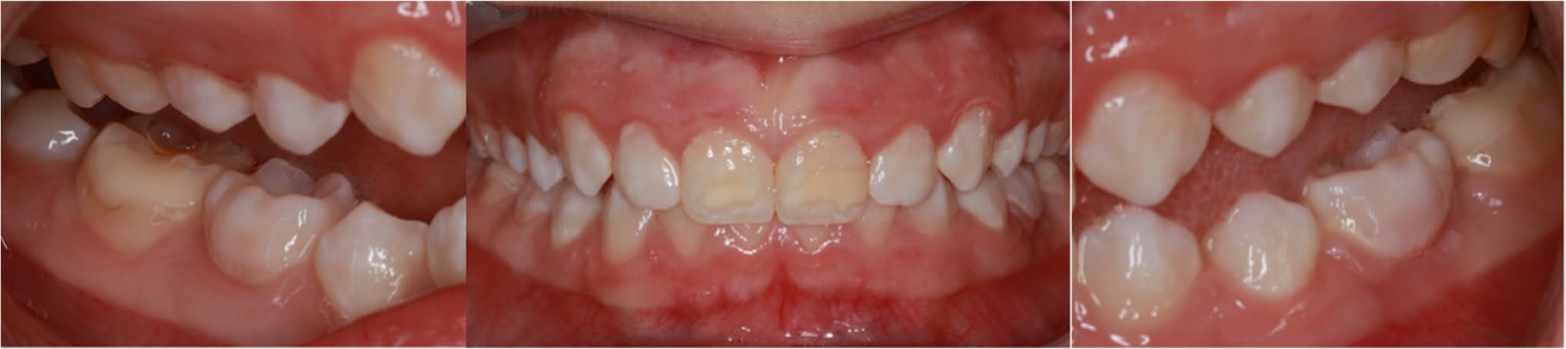
Intraoral pictures of a 9-year-old girl with PKU. MIH (molar incisor mineralization) is diagnosed. Teeth 36 and 46 were restored with partial crowns

Most enamel defects in patients with PKU, in our study, can be classified as “Molar Incisor Hypomineralization” (MIH) (Fig. 5). The definition of MIH has been implemented to characterize demarcated, qualitative enamel defects with an occasional post-eruptive breakdown of the soft and porous enamel. One or more first permanent molars with or without permanent incisor involvement are affected [20, 45].

This study and the 5^th^ German Oral Health Study (GOHS) clustered findings with MIH occurred. In the 5^th^ GOHS, 28.7% of 12-year-old children (random sample) had at least one first permanent molar with MIH signs [46]. In our study, no upper age limit was settled. Our DDE value for the siblings (21.4%) and the control group (19.0%) was slightly lower than the one of the GOHS group (28.7%). In contrast, patients with PKU have an enormously higher DDE involvement of 69%. Since the etiology of MIH/DDE with the underlying causative mechanism is inconclusive [17, 18], this increased DDE value in patients with PKU is of particular interest and may help in causal research.

DDE can have a substantial effect on oral health because it can be associated with tooth sensitivity [45], increased caries susceptibility [47], altered occlusal function, and esthetic appearance [17].

Therefore, exploring the etiology and pathogenesis of MIH/DDE is crucial. Subsequently, future studies should investigate other exposure factors of newborns with PKU during the amelogenesis of the first molars and incisors.

The increased caries prevalence in patients with PKU can be associated with high carbohydrate intake. Moreover, elevated DDE levels lead to more susceptible teeth [48]. The higher incidence of caries and periodontal disease in patients with PKU could also be attributed to the focus of parental and later own attitudes on maintaining normal general health, thus neglecting oral hygiene [26].

To disseminate this knowledge, the complete European guidelines of PKU [10] should also include the dental team under the item “Metabolic Team and Transition,” implementing prevention and standardized dental care.

Caregivers and medical practitioners involved in treating patients with PKU should be aware of these patients' increased dental risk, refer them early for the first dental appointment, and encourage dental education.

The protein substitutes of the PHE-restricted diet of the PKU patients should be mixed with low–sugar drinks.

Nutritional counseling, fluoridation, oral hygiene instructions, and regular professional dental cleaning should be implemented in the caries prevention program. The dentist should adjust the recall intervals throughout life according to the patient's caries risk.

We recommend these measures even though there is no clear evidence in our study that diet is the cause of dental disease.

## Conclusion

In this study, patients showed a higher risk of developing caries, periodontitis, and DDE than healthy individuals. The siblings had significantly fewer enamel defects but no significant differences in caries and periodontal parameters compared to the PKU patients.

The increased PCR (plaque accumulation) and GBI (gingival inflammation) were significant predictors of higher periodontitis risk. No questionnaire-based predictors were substantial for the increased risk of caries and DDE.

The diet of patients with PKU differs significantly from healthy individuals regarding the quantity and frequency of sweet food intake. Additionally, the PHE-free protein substitutes several times a day and often mixed with sweet drinks can promote a higher caries risk. However, a causal statement is complex to be provided, given the methodology used.

Therefore, better prevention instruments and implementations are needed to optimize dental care and improve long-term outcomes.

Further research on the etiologic factors that lead to the increased risk of DDE/MIH in PKU patients would elucidate the genetic and environmental factors contributing to enamel demineralization.

## Data Availability

The data presented in this study are available on reasonable request from the corresponding author.
